# Co-Incorporation of Green Manure and Rice Straw Increases Rice Yield and Nutrient Utilization

**DOI:** 10.3390/plants14111678

**Published:** 2025-05-30

**Authors:** Cuilan Wei, Bingshuai Cao, Songjuan Gao, Hao Liang

**Affiliations:** 1 College of Environment and Ecology, Jiangsu Open University, Nanjing 210036, China; weicl@jsou.edu.cn; 2 Nanjing Institute of Environmental Sciences, Ministry of Ecology and Environment, Nanjing 210042, China; 3 College of Resources and Environmental Sciences, Nanjing Agricultural University, Nanjing 210095, China; gaosongjuan@njau.edu.cn; 4 College of Geography and Remote Sensing, Hohai University, Nanjing 210098, China; haoliang@hhu.edu.cn

**Keywords:** milk vetch, rice straw return, soil nitrogen fertility, nutrient absorption

## Abstract

The co-incorporation of green manure and rice straw is commonly used to increase rice yield and improve soil fertility in paddy fields. However, the effects on nutrient uptake and utilization of rice under the synergistic interaction mechanism in the Taihu Plain of the Yangtze River Delta remain unclear. Based on field experiments, this study investigated the effects of green manure with rice straw return (GMS) under different nitrogen (N) fertilization rates on rice yield, nutrient use efficiency, and soil fertility. The results revealed that green manuring significantly increased rice yield while improving the uptakes and use efficiencies of N, phosphorus (P) and potassium (K). Green manure (GM) with 40% N fertilizer reduction (GM_N60) maintained the grain and straw yields and nutrient uptakes compared to winter fallow with 100% conventional N application (WF_N100). The N recovery efficiency in GM_N60 reached 45.52%, increasing by 41.26% compared to WF_N100. Rice yield and K uptake in the GMS with 40% N fertilizer reduction treatment (GMS_N60) was 10,058 and 15.41 kg/hm^2^, increasing by 14.43% and 9.43% compared to winter fallow with rice straw return and 100% conventional N (WFS_N100). The N, P and K agronomic efficiencies in GMS_N60 increased by 77.04%, 50.22%, and 50.22% compared to WFS_N100, respectively. These findings indicate that rice straw return enhances the fertilizer-saving and yield-increasing effects of GM, promotes rice K uptake and improves P and K use efficiencies. The GM treatment increased the soil organic matter (SOM), total potassium (TK), ammonium nitrogen (NH_4_^+^-N) and nitrate nitrogen (NO_3_^−^-N) contents. Among the soil fertility indicators, TK and SOM were the most important factors influencing rice yield and N uptake. In conclusion, GMS can maintain or increase rice yield with 40% N fertilizer reduction, improve nutrient use efficiencies, and increase the reuse of rice straw, thereby supporting green and efficient rice production in the southern Jiangsu paddy area.

## 1. Introduction

China is the world’s largest rice producer and consumer [1]. In the past three decades, the fertilizer application rate of rice in China has increased by more than 55%, but the single-yield rate has stagnated at 6–7 t/hm^2^ [2]. China’s cropping systems have surpassed the nitrogen (N) optimization threshold, where marginal yield gains exhibit progressive decline under conventional fertilization regimes [3,4]. Blind high-N fertilization or unreasonable fertilization not only reduces N use efficiency and wastes resources but also causes a series of ecological and environmental problems, such as soil acidification, water eutrophication, and greenhouse gas emissions [5,6,7]. Therefore, the rational application of chemical fertilizers, especially N fertilizers, is necessary for the development of green agriculture and stable high-yield rice production.

Leguminous green manures can input large amounts of N into agroecosystems via biological N fixation [8]. Leguminous green manure (GM) cultivation facilitates nutrient sequestration [9,10,11], and after incorporation, nutrients are released for subsequent crops [12]. Milk vetch (*Astragalus sinicus* L.), which is a common leguminous green manure in southern China, can maintain rice yield and improve soil fertility even with a 20–40% N fertilizer reduction [10,13,14,15].

Rice straw contains N, phosphorus (P), potassium (K), soil organic carbon (SOC), etc. Returning it to the field can replenish nutrients and stabilize soil carbon [16]. However, due to its high C/N ratio (50–70), microbial decomposition of crop straw requires substantial N uptake, consequently creating N competition with the main crop [17,18,19,20,21]. Leguminous green manures, with high N contents and low C/N ratios, decompose quickly. However, in practice, rice is transplanted two weeks after GM incorporation, and the optimal nutrient absorption period for young rice with low N demand is lost, resulting in nutrient loss [22].

Researchers have proposed the combined utilization of GM and rice straw to optimize the C/N ratio, thereby overcoming the limitations associated with their individual applications [23,24]. Studies have shown that incorporating both leguminous and non-leguminous crop residues enhances straw carbon and soil organic matter mineralization and increases microbial biomass [25,26,27]. In addition, the co-utilization of milk vetch and rice straw with 20% N fertilizer reduction maintains early rice yield and increases N uptake [28]. The co-incorporation of GM and rice straw serves as an effective strategy to simultaneously increase rice productivity and improve soil fertility. However, research on the co-utilization of milk vetch and rice straw in southern Jiangsu rice-growing areas is limited. We hypothesize that co-incorporation of milk vetch (low C/N ratio) and rice straw (high C/N ratio) optimizes the composite C/N ratio, synchronizes nutrient release with rice demand via microbial-mediated decomposition, and thereby enhances N, P, and K uptake efficiencies under reduced fertilizer regimes. This study, based on a field experiment in Lishui District, Jiangsu province, evaluates the effects of co-incorporation of milk vetch and rice straw with reduced N fertilization on rice yield, nutrient uptake efficiency, and soil fertility, aiming to clarify the potential of this practice to enhance rice productivity in the Taihu Plain of the Yangtze River Delta while providing a novel approach for sustainable straw utilization.

## 2. Materials and Methods

### 2.1. Experimental Site

**Figure 1:**
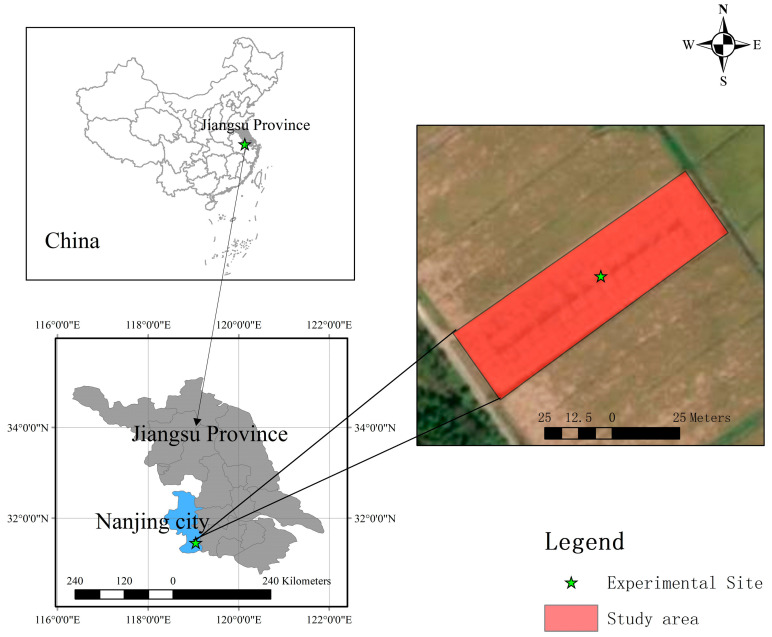
Schematic diagram of the experimental site.

The experiment was carried out in Jingqiao town, Lishui District, Nanjing city, Jiangsu province (31°45′69″ N, 119°05′45″ E) (Figure 1). The site is located in the Taihu Plain of the Yangtze River Delta, a typical rice-wheat rotation area with a subtropical monsoon climate. The annual average temperature is 15.5 °C, the annual sunshine duration is 2146 h, and the annual precipitation exceeds 1000 mm, mainly in spring and summer. The average frost-free period is 257 days. The tested soil type was yellow-brown. Prior to the experiment, the topsoil (0–20 cm) organic carbon content was 25.73 g·kg^−1^, the pH (1:2.5, soil/water) was 5.96, the total N content was 1.47 g·kg^−1^, and the inorganic N content was 8.67 g·kg^−1^.

### 2.2. Experimental Design and Implementation

The field experiment started in 2020 with 12 treatments: (1) WF_N0: winter fallow without rice straw return + no N fertilizer; (2) GM_N0: green manure (milk vetch) without rice straw return + no N fertilizer; (3) WFS_N0: winter fallow with rice straw return + no N fertilizer; (4) GMS_N0: green manure (milk vetch) with rice straw return + no N fertilizer; (5) WF_N60: winter fallow without rice straw + 60% conventional N fertilizer; (6) GM_N60: green manure (milk vetch) without rice straw return + 60% conventional N fertilizer; (7) WFS_N60: winter fallow with rice straw return + 60% conventional N fertilizer; (8) GMS_N60: green manure (milk vetch) with rice straw return + 60% conventional N fertilizer; (9) WF_N100: winter fallow without rice straw + 100% conventional N fertilizer; (10) GM_N100: green manure (milk vetch) without rice straw return + 100% conventional N fertilizer; (11) WFS_N100: winter fallow with rice straw return + 100% conventional N fertilizer; and (12) GMS_N100: green manure (milk vetch) with rice straw return + 100% conventional N fertilizer. A complete control without N, P, or K fertilizers was used for the calculation of fertilizer efficiency. Each treatment was repeated four times in a 20 m^2^ plot (4 m × 5 m) with a completely randomized block design.

The milk vetch variety used was “Yijiangzi”, and the midseason rice variety was “Jingliangyou Hua Zhan”, which are the most cultivated varieties locally. Rice plants were transplanted in mid-June and harvested at the end of September each year. Milk vetch was sown in early October and turned under in mid-April the following year during full bloom. Milk vetch and rice straw were chopped finely and subsequently returned to the topsoil. The characteristics of milk vetch and rice straw are shown in Table 1. The N fertilizer used was urea (46% N), the P fertilizer was calcium superphosphate (12% P_2_O_5_), and the K fertilizer was potassium chloride (60% K_2_O). The N fertilizer was split into basal and tillering fertilizers (1:1), while the P and K fertilizers were all applied as basal fertilizers. The field management and fertilizer application rates for each treatment are shown in Table 2.

### 2.3. Sample Collection and Index Determination

Rice yield was measured at maturity from 2021 to 2024, with individual harvesting per plot. In 2023, plant and soil samples were collected at rice maturity. Three random rice plants were taken from each plot. After soil–root separation and washing, the plants were divided into aboveground and belowground parts. Plant samples were deactivated at 105 °C for 30 min to halt enzymatic activity, followed by drying to constant weight at 70 °C. For mature plants, grains and straw were separated. The plant samples were digested with concentrated sulfuric acid and hydrogen peroxide. The total N content was determined by the Kjeldahl method, the total P content was determined via the vanadomolybdophosphoric acid colorimetric method, and the total K content was determined via flame photometry [29].

The soil samples were stored at 4 °C for mineral nitrogen determination. The remaining samples were air-dried, ground, and sieved for other soil property tests. The specific methods used were as follows: Soil organic matter (SOM) and total nitrogen (TN) were measured with an elemental analyzer (Flash Smart, Thermo Fisher Scientific, Waltham, MA, USA). Soil pH was determined by the potential method at a water–soil ratio of 2.5:1. Soil available phosphorus (AP) was extracted with 0.5 mol/L sodium bicarbonate and determined by the molybdenum–antimony–scandium colorimetric method. Soil available potassium (AK) was extracted with 1 mol/L ammonium acetate and measured by atomic absorption spectrometry. Mineral N was extracted with 2 mol/L potassium chloride and analyzed using a continuous flow analyzer (SAN++, Skalar, Breda, The Netherlands). Dissolved organic carbon (DOC) was determined by shaking ultrapure water with a 5:1 water-to-soil ratio, centrifuging, filtering the supernatant through a 0.45 μm membrane, and analyzing with a total organic carbon analyzer (TOC-L CPH, Shimadzu, Kyoto, Japan) [29]. The calibration curves and recovery rate determinations for each tested indicator met the testing requirements.

### 2.4. Data Analysis

The following formulas were used to calculate the absorption of N, P, and K by grains and plants, as well as the fertilizer efficiency rate, the fertilizer agronomic efficiency, the fertilizer partial productivity, and the fertilizer harvest index [13]:

*GA* (N (P, K) grain accumulation) (kg/hm^2^) = Grain (N (P, K)) content × *GY* (1)

*PA* (N (P, K) plant accumulation) (kg/hm^2^) = Straw (N (P, K)) content × *SDY* + *GA* (2)

FREA (N (P, K)) fertilizer recovery efficiency) (%) = (*F* − *F*_0_)/*F_T_* ∗ 100% (3)

FAE (N (P, K) fertilizer agronomic efficiency) (kg/kg) = (*G* − *G*_0_)/*F_T_* (4)

FPP (N (P, K) fertilizer partial productivity) (kg/kg) = *G_T_*/*F_T_* (5)

FHI (N (P, K) fertilizer harvest index) (%) = *GA*/*PA* ∗ 100% (6)

where *GA* is the grain accumulation of N (P, K), *GY* is the grain yield, *PA* is the plant accumulation of N (P, K), *SDY* is the straw dry weight, FRE is the fertilizer recovery efficiency of N (P, K), *F* is the total N (P, K) in the fertilized treatments, *F*_0_ is the total N (P, K) in the unfertilized control, *F_T_* is the total fertilizer N (P, K) applied, FAE is the fertilizer agronomic efficiency of N (P, K), *G* is the grain yield in the fertilized treatments, *G_0_* is the grain yield in the unfertilized control, FPP is the fertilizer partial productivity of N (P, K), *G_T_* is the total yield in the fertilized treatments, and FHI is the fertilizer harvest index of N (P, K).

The effects of different treatments on rice yield, N, P, and K accumulation, and soil properties were analyzed using SAS 8.1. SPSS 24.0 was used for variance analysis, followed by multiple comparisons using the least significant difference (LSD) test at *p* < 0.05. All figures were created using Origin 2018 (Version 9.0, Learning Edition). The impact of soil fertility on rice yield and nutrient absorption was evaluated using random forest analysis, which was performed by constructing an ensemble of 500 decision trees using bootstrap sampling and random feature selection at each node split using R (v. 4.2.2). In random forest regression models, %IncMSE (percentage increase in mean squared error) is used to quantify how much the model’s prediction error increases when a given variable is randomly permuted. A higher %IncMSE indicates that the variable contributes more to predictive accuracy, as its removal leads to a larger degradation in model performance.

## 3. Results and Analysis

### 3.1. Rice and Straw Yield Under the Co-Utilization of Milk Vetch and Rice Straw

**Figure 2:**
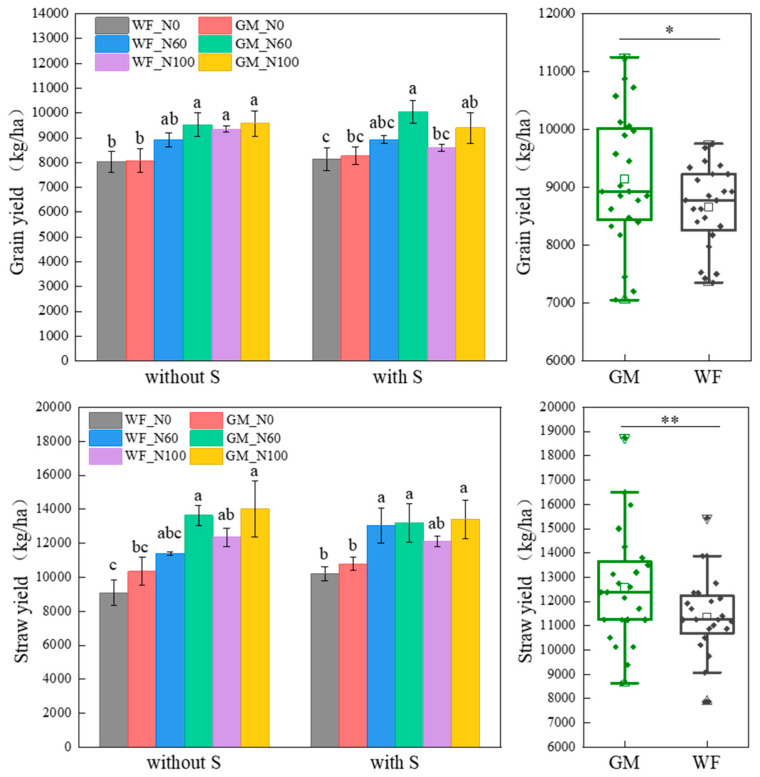
Average rice and straw yield under different treatments. Note: Without S and with S represent without rice straw return and with rice straw return, respectively. WF_N0, WF_N60, and WF_N100 represent winter fallow with 0%, 60%, and 100% conventional N fertilizer, respectively. GM_N0, GM_N60, and GM_N100 represent green manuring with 0%, 60%, and 100% conventional N fertilizer, respectively (*n* = 4). GM represents the combination of all 6 treatments with green manure, and WF represents the combination of all 6 treatments without green manure (*n* = 24). Different letters (a, b, c) indicate significant differences (*p* < 0.05). In the box figures, the solid line and dot within each box represent the median and mean values, respectively. The top and bottom edges represent the 75th and 25th percentiles, respectively; the top and bottom error bars represent the 95th and 5th percentiles, respectively; and the top and bottom triangles represent the 99th and 1st percentiles, respectively. * represents *p* < 0.05; ** represents *p* < 0.01.

The multiyear average yields revealed that at the same N level, the GM treatments (with or without straw return) presented higher rice and straw yields than that in WF treatments. Compared to WF, the average rice and straw yields in GM treatments increased by 5.34% and 9.60% (*p* < 0.05), respectively (Figure 2). Compared to WF_N100, rice yield in GM_N60 and GM_N100 treatments increased by 1.92% and 2.44%, while straw yield increased by 9.52% and 12.06%, respectively. Under winter fallow conditions, the rice and straw yields increased with increasing N application (Figure 2). Compared to WFS_N100, rice yield in GMS_N60 and GMS_N100 treatments increased by 14.44% and 8.36%, while straw yield increased by 8.14% and 9.56%, respectively. Under winter fallow without rice straw returning conditions, the rice and straw yields increased with increasing N application (Figure 2). Under winter fallow with rice straw returning conditions, the highest rice and straw yields occurred in the WFS_N60 treatment (Figure 2).

### 3.2. N Absorption and Utilization of the Co-Utilization of Milk Vetch and Rice Straw

**Figure 3:**
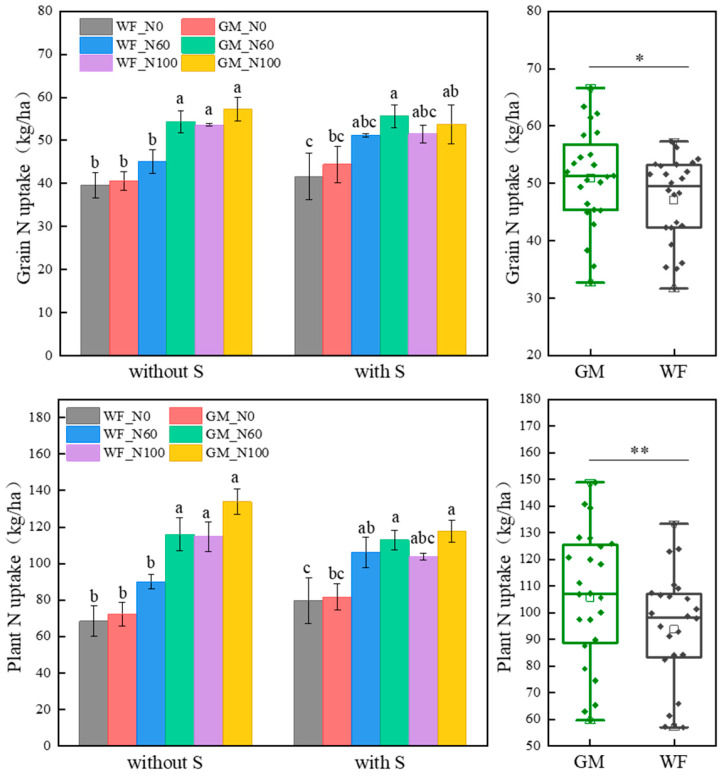
Grain and plant N accumulation under different treatments. Note: Without S and with S represent without rice straw return and with rice straw return, respectively. WF_N0, WF_N60, and WF_N100 represent winter fallow with 0%, 60%, and 100% conventional N fertilizer, respectively. GM_N0, GM_N60, and GM_N100 represent green manuring with 0%, 60%, and 100% conventional N fertilizer, respectively (*n* = 4). GM represents the combination of all 6 treatments with green manure, and WF represents the combination of all 6 treatments without green manure (*n* = 24). Different letters (a, b, c) indicate significant differences (*p* < 0.05). In the box figures, the solid line and dot within each box represent the median and mean values, respectively. The top and bottom edges represent the 75th and 25th percentiles, respectively; the top and bottom error bars represent the 95th and 5th percentiles, respectively; and the top and bottom triangles represent the 99th and 1st percentiles, respectively. * represents *p* < 0.05; ** represents *p* < 0.01.

The average N accumulation of rice grains and plants in GM treatment increased by 7.58% and 11.33% (*p* < 0.05) compared to WF treatment, respectively (Figure 3).

Without rice straw returning, the grain N uptake in GM treatments at N60 and N100 levels increased by 16.92% (*p* < 0.05) and 6.36% compared to WF treatments, and the whole-plant N uptake increased by 22.38% (*p* < 0.05) and 14.30%, respectively. Compared to WF_N100, the grain and whole-plant N accumulation in GM_N60 treatment increased by 1.37% and 0.98%, respectively.

Under rice straw returning conditions, the grain N uptake in GMS treatments at N0 and N60 levels increased by 6.27% and 8.03% compared to WFS treatments, and the whole-plant N uptake increased by 2.50% and 6.03%, respectively. There were no significant differences in grain or whole-plant N uptake between GMS and WFS treatments at the N100 level. Compared to WFS_N100, GMS_N60 increased the grain and whole-plant N uptake by 7.37% and 8.13%, respectively.

Compared to WF, the average N fertilizer recovery efficiency, agronomic efficiency, and partial productivity in GM treatments increased by 28.69%, 37.21%, and 7.58% (*p* < 0.05), respectively (Table 3). The N fertilizer recovery efficiency was highest in GM_N60 treatment. Without rice straw returning, the N fertilizer recovery efficiency in GM_N60 treatment increased by 41.25% and 46.51% compared to WF_N100 and WF_N60 treatments, and the N fertilizer partial productivity in GM_N60 treatment increased by 41.16% and 2.44% (*p* < 0.05) compared to WF_N100 and GM_N100 treatments, respectively (Table 3). Under rice straw returning conditions, the N fertilizer recovery efficiency in GMS_N60 treatment increased by 50.68% (*p* < 0.05) compared to WFS_N100. Compared to WFS_N60, WFS_N100, and GMS_N100 treatments, the N fertilizer agronomic efficiency in GMS_N60 treatment increased by 48.18%, 77.04%, and 56.95%, and the partial productivity increased by 11.27%, 48.66%, and 43.96% (*p* < 0.05), respectively (Table 3).

### 3.3. P Absorption and Utilization of Rice Under the Co-Utilization of Milk Vetch and Rice Straw

**Figure 4:**
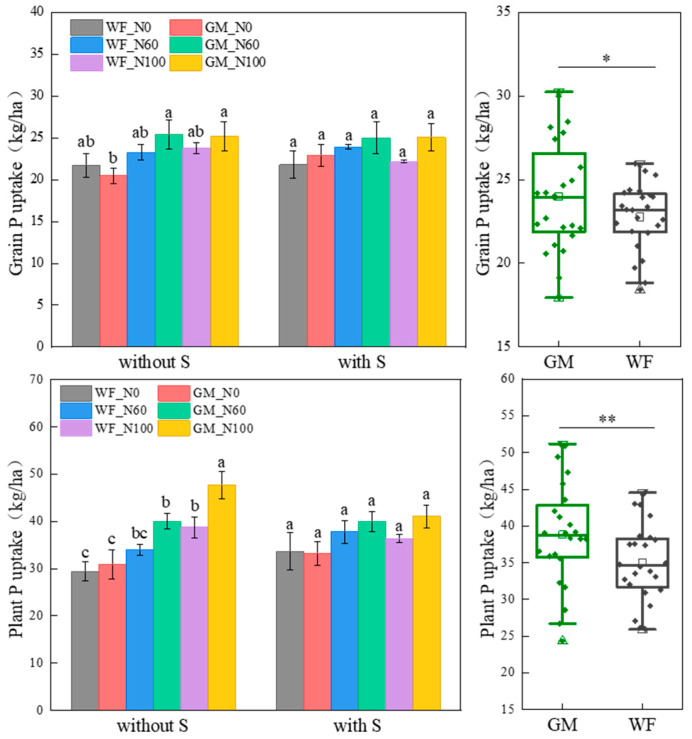
Grain and plant P accumulation under different treatments. Note: Without S and with S represent without rice straw return and with rice straw return, respectively. WF_N0, WF_N60, and WF_N100 represent winter fallow with 0%, 60%, and 100% conventional N fertilizer, respectively. GM_N0, GM_N60, and GM_N100 represent green manuring with 0%, 60%, and 100% conventional N fertilizer, respectively (*n* = 4). GM represents the combination of all 6 treatments with green manure, and WF represents the combination of all 6 treatments without green manure (*n* = 24). Different letters (a, b, c) indicate significant differences (*p* < 0.05). In the box figures, the solid line and dot within each box represent the median and mean values, respectively. The top and bottom edges represent the 75th and 25th percentiles, respectively; the top and bottom error bars represent the 95th and 5th percentiles, respectively; and the top and bottom triangles represent the 99th and 1st percentiles, respectively. * represents *p* < 0.05; ** represents *p* < 0.01.

The average P accumulation of rice grains and plants in GM treatment increased by 7.58% and 11.33% (*p* < 0.05) compared to WF treatment, respectively. Without rice straw returning, the grain P accumulation in GM_N60 and GM_N100 treatments increased by 6.84% and 5.77% compared to WF_N100 treatment, while the whole-plant P accumulation increased by 3.47% and 23.18% (*p* < 0.05), respectively. At the N0, N60, and N100 levels, the whole-plant P accumulation in GM treatment was 4.97%, 15.01%, and 18.78% higher than that in WF treatment, respectively (Figure 4).

Compared to WF treatment, the P fertilizer recovery efficiency, agronomic efficiency, and partial productivity in GM treatment significantly increased by 32.69%, 27.86%, and 7.14% (*p* < 0.05), respectively (Table 4). Without rice straw returning, GM treatment increased P fertilizer use efficiency at the same N level compared to WF. Compared to WF treatments, the P fertilizer recovery efficiencies in GM treatments increased by 48.81%, 41.21%, and 40.32% at N0, N60 and N100 levels, respectively. The P fertilizer recovery efficiency, agronomic efficiency, partial productivity, and harvest index in GM_N60 treatment increased by 9.23%, 7.77%, 1.92%, and 2.97% compared to WF_N100 treatment, respectively. Under rice straw returning conditions, the P fertilizer agronomic efficiency in GMS_N60 treatment increased by 59.08%, 39.20%, and 50.22% (*p* < 0.05) compared to WFS_N0, WFS_N60, and WFS_N100 treatments, while the P fertilizer partial productivity increased by 16.98%, 11.27%, and 14.44% (*p* < 0.05), respectively (Table 4).

### 3.4. K Absorption and Utilization of Rice Under the Co-Utilization of Milk Vetch and Rice Straw

**Figure 5:**
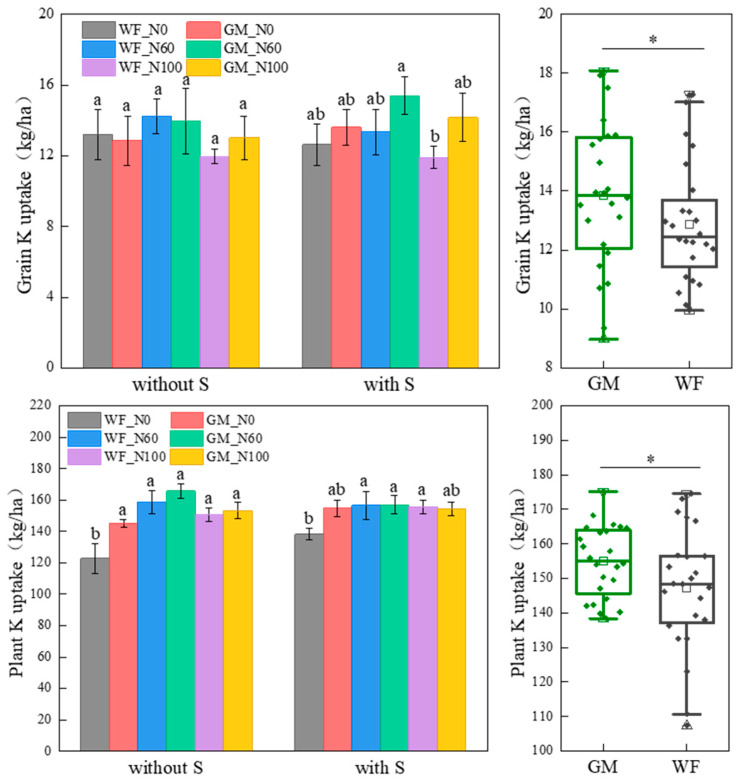
Grain and plant K accumulation under different treatments. Note: Without S and with S represent without rice straw return and with rice straw return, respectively. WF_N0, WF_N60, and WF_N100 represent winter fallow with 0%, 60%, and 100% conventional N fertilizer, respectively. GM_N0, GM_N60, and GM_N100 represent green manuring with 0%, 60%, and 100% conventional N fertilizer, respectively (*n* = 4). GM represents the combination of all 6 treatments with green manure, and WF represents the combination of all 6 treatments without green manure (*n* = 24). Different letters (a, b) indicate significant differences (*p* < 0.05). In the box figures, the solid line and dot within each box represent the median and mean values, respectively. The top and bottom edges represent the 75th and 25th percentiles, respectively; the top and bottom error bars represent the 95th and 5th percentiles, respectively; and the top and bottom triangles represent the 99th and 1st percentiles, respectively. * represents *p* < 0.05.

The average K accumulation of rice grains and plants in GM treatment increased by 6.99% and 5.12% (*p* < 0.05) compared to WF treatment, respectively. Without rice straw returning, there were no differences in grain K accumulation among the treatments, whereas WF_N0 resulted in significantly lower whole-plant K accumulation than the other treatments did. Among them, the whole-plant K accumulation in GM_N0 treatment increased by 15.36% (*p* < 0.05) compared to WF_N0 treatment. Under rice straw returning conditions, the grain K accumulation in GMS_N60 treatment increased by 22.72% (*p* < 0.05) compared to WFS_N100 treatment, and the whole-plant K accumulation increased by 11.99% (*p* < 0.05) compared to WFS_N0 treatment (Figure 5).

In GM treatment, the average K fertilizer recovery efficiency, agronomic efficiency, and partial productivity were 3.65%, 27.86%, and 7.14% (*p* < 0.05) higher than those in WF treatment, respectively (Table 5). Without rice straw returning, the K fertilizer recovery efficiency in GM_N0, GM_N60, and GM_N100 treatments increased by 46.78%, 62.94%, and 54.75% (*p* < 0.05) compared to WF_N0 treatment, respectively. The K fertilizer agronomic efficiency in GM_N60 and GM_N100 treatments increased by 63.88% and 64.63% (*p* < 0.05) compared to WF_N0 treatment. The K fertilizer partial productivity in GM_N60 and GM_N100 treatments increased by 15.29% and 15.73% (*p* < 0.05) compared to GM_N0 treatment. There were no significant differences in the K harvest indices among the treatments. Under rice straw returning conditions, the K fertilizer recovery efficiency was the highest in GMS_N60 treatment, with an increase of 31.60% (*p* < 0.05) compared to WFS_N0 treatment. Compared to WFS_N0, WFS_N60, and WFS_N100, the K fertilizer agronomic efficiency in GMS_N60 treatment increased by 31.60%, 0.80%, and 2.20% (*p* < 0.05), and the K fertilizer partial productivity increased by 66.43%, 39.20%, and 50.22% (*p* < 0.05), respectively. There were no significant differences in the K harvest indices among the treatments.

### 3.5. Impact of Co-Utilization of Milk Vetch and Rice Straw on Soil Fertility

The differences in soil nutrient content among the treatments were minor. Compared to WF treatment, the average SOM, total phosphorus (TP), ammonium nitrogen (NH_4_^+^-N) and nitrate nitrogen (NO_3_^−^-N) contents in GM treatment increased by 2.12%, 6.51%, 23.77%, and 14.15%, respectively. The SOM content in GMS treatments showed an increasing trend compared to GM and WFS treatments at the same N level, indicating a synergistic effect on SOM under the co-incorporation of green manure and rice straw. As N application increased, the SOM content increased, with average contents of 21.45, 22.12, and 22.44 g/kg under N0, N60, and N100, respectively. In contrast, TN and NO_3_^−^-N decreased, whereas NH_4_^+^-N increased (Table 6). There were no significant differences in SOM, NO_3_^−^-N, AP, or AK among the treatments. WF_N0 treatment had the highest TN content, which was 19.01–21.13% greater than that in the other treatments. GMS_N0 treatment had the highest TP content, with an increase of 14.58% (*p* < 0.05) compared to GM_N60 treatment. The TK content in GMS_N0 treatment was significantly higher than that in the other treatments (except WFS_N0) by 5.51–40.34%. The NH_4_^+^-N content in GM_N100 treatment was 45.45–68.18% higher than that in the other treatments (Table 6).

### 3.6. Impact of Soil Fertility on Rice Yield and Nutrient Absorption Under Co-Utilization of Milk Vetch and Rice Straw

**Figure 6:**
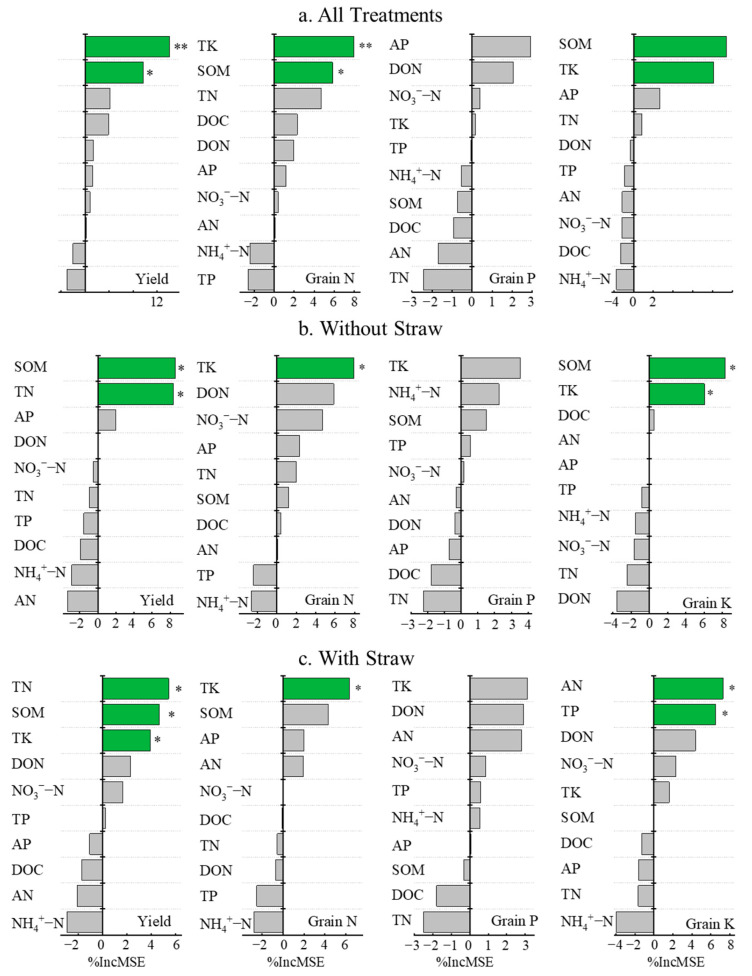
Effects of soil properties and different treatment factors on rice yield and grain N, P, and K content. Note: %IncMSE represents percentage increase in mean squared error. TK, SOM, NO_3_^−^-N, TN, AP, DON, AN, TP, DOC, and NH_4_^+^-N represent soil total potassium, soil organic matter, nitrate nitrogen, total nitrogen, available phosphorus, dissolved organic nitrogen available nitrogen, total phosphorus, dissolved organic carbon, and ammonium nitrogen, respectively. * represents *p* < 0.05; ** represents *p* < 0.01; Green indicates significant differences, while gray indicates no significant differences.

The random forest analysis revealed that when the GM and rice straw factors were considered together, among the soil fertility indicators, the TK and SOM significantly affected the rice yield and the grain N and grain K contents. The contribution rates of TK and SOM to rice yield were 14.01% (*p* < 0.01) and 9.68% (*p* < 0.05); those to grain N content were 7.94% (*p* < 0.01) and 5.85% (*p* < 0.05); and those to grain P content were 8.14% and 9.40% (*p* < 0.05). No soil properties significantly affected the grain P content (Figure 6). Without rice straw returning, the SOM and TN significantly affected the rice yield, with contribution rates of 8.57% and 8.37% (*p* < 0.05), respectively. The TK affected the total grain N content significantly with a contribution rate of 7.94% (*p* < 0.01). No soil properties significantly affected the grain P content. SOM and TK affected the grain K content significantly with contribution rates of 8.31% and 6.04% (*p* < 0.05), respectively (Figure 6). Under rice straw returning conditions, the TN, SOM, and TP contents significantly affected the rice yield, with contribution rates of 5.45%, 4.71%, and 3.97% (*p* < 0.05), respectively. The contribution rate of TK to the total grain N content was 6.33% (*p* < 0.05). No soil properties significantly affect the grain P content. The contribution rates of AN and TP to the grain K content were 7.29% and 6.49% (*p* < 0.05), respectively (Figure 6).

**Figure 7:**
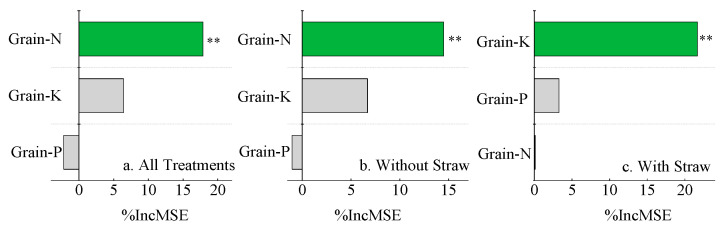
Contributions of grain N, P, K content and different treatment factors to rice yield. Note: %IncMSE represents percentage increase in mean squared error. Grain-N, Grain-P, and Grain-K represent the content of grain N, P, and K, respectively. ** represents *p* < 0.01; Green indicates significant differences, while gray indicates no significant differences.

Further analysis was performed on the impacts of the grain N, P, and K contents on the rice yield. In the treatments with both GM and straw and without straw, the grain N content had the greatest effect on the rice yield, at 17.87% and 14.49% (*p* < 0.01), respectively. Among the treatments with straw, the grain K content most significantly affected the rice yield, at 21.67% (*p* < 0.01) (Figure 7).

## 4. Discussion

### 4.1. Impact of Milk Vetch Combined with Rice Straw on Rice Yield and Nutrient Absorption

The incorporation of green manure (GM) and the rice straw return can promote rice growth and increase yield and nutrient accumulation while reducing fertilizer use [30]. Compared with their separate application, the combined application of milk vetch and the rice straw further increases grain yield [31]. In Jiangxi, the co-incorporation of GM and rice straw advanced early rice tillering by five days, increasing yield indicators such as the stem-tiller number and tillering spike rate, thereby increasing early and late rice yields by 5.39% and 2.56%. It also improved nutrient absorption, chlorophyll content, photosynthesis, and grain quality [32,33]. In this study, in the southern Jiangsu paddy fields, the co-incorporation of GM and rice straw maintained a rice yield with 40% N fertilizer reduction, which aligns with previous research [31,32,33]. This suggests that it is a viable technique for improving rice production in this area. Planting milk vetch in winter fallow rice fields increase the soil N input via symbiotic N fixation with rhizobia and reduces N leaching [28]. After incorporation, it provides nutrients for the next crop [34]. Compared with conventional fertilization, the application of milk vetch and straw with a 0–40% reduction in N fertilizer maintained early rice nutrient absorption in Lishui District. This finding is consistent with those of Wang et al. [35] that 40% nitrogen substitution with milk vetch increased rice nitrogen uptake [35]. Field measurements indicate that milk vetch produces an average fresh biomass yield of 22500 kg/hm^2^, with biological N fixation ranging from 26.5 to 54.8 kg/hm^2^. This represents significant N substitution potential, corresponding to 17.7–36.5% of conventional rice fertilization requirements. [36]. Adequate N also promotes P and K absorption [37,38]. Milk vetch combined with rice straw return resulted in greater nutrient absorption than GM alone, indicating greater nutrient release. This combination avoids issues from individual application, synchronizes nutrient release with rice uptake, and lowers costs. When rice straw is coapplied, its active carbon promotes soil microbial immobilization of legume-derived N, reducing competition and facilitating temporally aligned N availability for crops [17,18,19,20,21,39]. Research indicates that co-application of rice straw and leguminous GM can affect the decomposition process of different straws and alter the decomposition pattern of single-crop straw [22,24]. The active organic carbon released by rice straw can promote the immobilization of N from legume straw by soil microbes, alleviating competition for soil N and ensuring slow and sustained N release for crop uptake, which increases N use efficiency and reduces nutrient loss [40,41,42,43]. The application of milk vetch reduces fertilizer inputs and increases N efficiency [44]. In this study, with a 40% reduction in N fertilizer, the N efficiency increased by 41.25% and 34.28% for milk vetch and combined rice straw return, respectively (Table 3). This occurred because the N fixation ability of milk vetch provides available N for rice [23]. At 40% N fertilizer reduction, the agronomic and partial N efficiencies were highest under the co-incorporation of milk vetch and the rice straw. Liu et al. (2017) reported similar increases in N efficiency under milk vetch with 20–40% N fertilizer reduction in central Henan [45]. Using milk vetch with reduced fertilizer enhances N absorption and soil residual N [46]. Compared with chemical nitrogen, the N released during milk vetch decomposition better matches rice N requirements, thus improving N use efficiency [47].

### 4.2. Effects of Soil Fertility Improvement by Combining Milk Vetch and Rice Straw Return on Rice Growth and Nutrient Absorption

Soil pH, TN, and SOM are key fertility indicators affecting rice growth and yield [48]. In this study, under 40% N reduction, the milk vetch–rice straw combination return treatment resulted in the highest TN and SOM. Previous studies have shown that rice straw return increases SOM (5.8–28.9%) and TN (26.0%) contents [49,50,51] and improves soil structure [52]. GM incorporation lowers the soil redox potential, releases organic acids, and activates AP and mineral K [53,54,55]. Combined milk vetch–rice straw return leverages the fertilization benefits of both components more comprehensively than individual application does [23,56]. Compared with the control, in the two-crop rice area of Hunan, five years of combined return increased TN and SOM by 12.93–22.41% and 16.57–29.00%, respectively [57]. This practice also improves soil properties, stabilizes the N supply, and increases soil organic carbon by 15.7–20.9% compared with individual applications [18]. An appropriate C/N ratio of mixed materials reduces the priming effect of leguminous GM and enhances the microbial utilization of non-leguminous straw, resulting in the formation of stable humus and increasing the SOM and stability [58]. There was a significant interaction effect between rice N and K uptake. This study revealed a significant synergistic effect between the soil total K content and rice N uptake and yield. Under the combined application of GM and rice straw, the TK contributed 7.94% to the grain N content and 14.01% to the rice yield, indicating that K plays a crucial role in optimizing N efficiency. In the straw-free treatments, the grain N content had a greater contribution to yield (14.49%) than the other factors, whereas the TK contributed 7.94% to the grain N content, further confirming the N-K interaction. In addition, rice straw return significantly altered soil nutrient availability and yield. In the straw-applied treatments, the grain K content contributed to yield by 21.67%, surpassing the other indicators, whereas the TN and TP contributions increased to 5.45% and 3.97%, respectively. These changes may be due to the release of organic carbon and mineral nutrients during straw decomposition, which promotes microbial activity, accelerates N and P mineralization, and directly supplements soil K. However, straw return may also cause competition for N due to its high C/N ratio, leading to temporary N immobilization and fluctuating N availability, which may explain the higher contribution of SOM and TN (8.57% and 8.37%) to yield in the straw-free treatments. While our study demonstrates clear patterns of improved N, P, and K uptake under co-utilization of milk vetch and rice straw, physiological properties enhanced by green manuring can also contribute to soil fertility improvement. The observed nutrient benefits may arise from synergistic interactions between root morphological adaptations, enhanced nutrient transporter activity, and microbial-mediated processes under green manuring [59]. For instance, the decomposition of milk vetch releases labile carbon and biologically fixed N [18], which could stimulate root proliferation and upgrade high-affinity transporters in rice [60]. Notably, co-incorporation likely modifies rhizosphere pH and redox conditions, further influencing nutrient bioavailability [9]. Future work should explicitly quantify these pathways, such as root exudate profiles or transporter gene expression, to disentangle their relative contributions.

Co-utilization of milk vetch and rice straw as an effective measure to enhance rice nutrient uptake and improve soil fertility has been widely adopted in southern China’s rice-growing regions. However, its environmental benefits still require further attention, including the prioritization of quantifying trade-offs between nutrient efficiency and greenhouse gas emissions (e.g., methane from anaerobic straw decomposition and nitrous oxide from N mineralization) while monitoring long-term soil health impacts. Additionally, socioeconomic assessments of labor costs for green manure cultivation and mechanized straw incorporation will be conducted to address adoption barriers in diverse agroecological zones. These investigations aim to refine GMS protocols for carbon-neutral rice production, ensuring alignment with global goals of soil health restoration and climate-resilient agriculture.

## 5. Conclusions

The application of green manure significantly enhanced rice yield. Compared to winter fallow, the co-incorporation of green manure and rice straw (GMS) maintained stable rice production while reducing N fertilizer input by 40%. Under the 60% conventional N application, GMS demonstrated superior N, P, and K uptake capacity, thereby improving nutrient use efficiency. Furthermore, GMS enhanced paddy soil fertility, with increased SOM, TK, and TN content identified as key drivers of yield improvement and nutrient accumulation. This study highlights that the co-incorporation of green manure and rice straw (GMS) with 40% nitrogen (N) reduction effectively enhances nutrient utilization efficiency, improves soil fertility, and sustains rice productivity, validating its potential as a sustainable alternative to conventional practices.

## Figures and Tables

**Table 1:** The characteristics of milk vetch and rice straw.

Treatment	C Content (g/kg)	N Content (g/kg)	P Content (g/kg)	C/N Ratio	C/P Ratio	N/P Ratio
GM	445.1 ± 2.4	32.0 ± 1.3	3.4 ± 0.2	13.9 ± 0.6	131.3 ± 6.3	9.4 ± 0.4
RS	423.2 ± 6.4	8.9 ± 0.1	2.1 ± 0.2	47.3 ± 0.5	204.8 ± 18.2	4.3 ± 0.4
GMS	429.4 ± 10.8	14.2 ± 0.5	2.4 ± 0.1	30.4 ± 1.4	182.0 ± 10.0	6.0 ± 0.4

Note: GM, RS and GMS represent green manure (milk vetch), rice straw, and green manure (milk vetch) with rice straw return, respectively.

**Table 2:** Field management and fertilizer application rates for each treatment.

Treament	Winter Crop	Rice Straw Return	N (kg/ha)	P_2_O_5_ (kg/ha)	K_2_O (kg/ha)
WF_N0	Winter fallow	no rice straw return	0	75	100
GM_N0	Green manure (milk vetch)	no rice straw return	0	75	100
WFS_N0	Winter fallow	rice straw return	0	75	100
GMS_N0	Green manure (milk vetch)	rice straw return	0	75	100
WF_N60	Winter fallow	no rice straw return	120	75	100
GM_N60	Green manure (milk vetch)	no rice straw return	120	75	100
WFS_N60	Winter fallow	rice straw return	120	75	100
GMS_N60	Green manure (milk vetch)	rice straw return	120	75	100
WF_N100	Winter fallow	no rice straw return	200	75	100
GM_N100	Green manure (milk vetch)	no rice straw return	200	75	100
WFS_N100	Winter fallow	rice straw return	200	75	100
GMS_N100	Green manure (milk vetch)	rice straw return	200	75	100

Note: WF, GM, WFS and GMS represent winter fallow without rice straw return, green manure (milk vetch) without rice straw return, winter fallow with rice straw return, and green manure (milk vetch) with rice straw return, respectively. N0, N60, and N100 represent no N fertilizer, 60% conventional N fertilizer and 100% conventional N fertilizer application, respectively.

**Table 3:** N fertilizer use efficiency under co-utilization of milk vetch and rice straw.

Treatment		N Fertilizer Recovery Efficiency (%)	N Fertilizer Agronomic Efficiency (kg/kg)	N Fertilizer Partial Productivity (kg/kg)	N Fertilizer Harvest Index (%)
without S	WF_N60	23.90 ± 3.32 b	10.16 ± 2.38 a	74.38 ± 2.38 a	50.16 ± 1.90 a
	GM_N60	45.52 ± 7.53 a	15.16 ± 3.91 a	79.38 ± 3.91 a	47.81 ± 4.57 a
	WF_N100	26.74 ± 4.06 b	8.18 ± 0.60 a	46.71 ± 0.60 b	47.35 ± 3.17 a
	GM_N100	36.32 ± 3.37 ab	9.35 ± 2.55 a	47.88 ± 2.55 b	42.94 ± 2.49 a
with S	WF_N60	37.32 ± 6.86 a	10.16 ± 1.35 b	74.38 ± 1.35 b	49.13 ± 3.86 a
	GM_N60	42.99 ± 4.31 a	19.60 ± 3.81 a	83.82 ± 3.81 a	49.72 ± 3.93 a
	WF_N100	21.20 ± 0.94 b	4.50 ± 0.72 b	43.03 ± 0.72 c	49.65 ± 1.40 a
	GM_N100	28.25 ± 3.02 ab	8.44 ± 3.04 b	46.97 ± 3.04 c	45.54 ± 2.58 a
GM		38.27 ± 2.79 **	13.14 ± 1.91 **	64.51 ± 4.68 **	46.50 ± 1.70 ^n.s.^
WF		27.29 ± 2.50	8.25 ± 0.88	59.62 ± 3.88	49.07 ± 1.26

Note: Data are presented as the mean ± standard error of 4 replicates. Different letters within the same column indicate significant differences (*p* < 0.05); ** represents *p* < 0.01; n.s. represent no significant difference (*p* > 0.05); The same applies below.

**Table 4:** P fertilizer use efficiency under co-utilization of milk vetch and rice straw.

Treatment		P Fertilizer Recovery Efficiency (%)	P Fertilizer Agronomic Efficiency (kg/kg)	P Fertilizer Partial Productivity (kg/kg)	P Fertilizer Harvest Index (%)
without S	WF_N0	11.92 ± 6.24 d	11.36 ± 5.64 b	106.92 ± 5.64 b	74.04 ± 2.22 a
	GM_N0	23.29 ± 6.70 cd	16.55 ± 4.52 ab	112.11 ± 4.52 ab	65.28 ± 3.27 ab
	WF_N60	26.18 ± 3.55 bcd	23.44 ± 3.81 ab	119.00 ± 3.81 ab	68.42 ± 1.82 ab
	GM_N60	44.53 ± 4.83 b	31.44 ± 6.26 a	127.00 ± 6.26 a	63.81 ± 4.88 abc
	WF_N100	40.43 ± 6.97 bc	29.00 ± 1.59 a	124.56 ± 1.59 a	61.91 ± 2.62 bc
	GM_N100	67.74 ± 8.84 a	32.11 ± 6.80 a	127.67 ± 6.80 a	53.27 ± 4.23 c
with S	WF_N0	31.75 ± 9.86 a	15.77 ± 5.57 b	111.33 ± 5.57 b	64.32 ± 2.16 a
	GM_N0	30.36 ± 3.78 a	19.44 ± 0.82 b	115.00 ± 0.82 b	68.62 ± 2.13 a
	WF_N60	37.67 ± 7.35 a	23.44 ± 2.16 b	119.00 ± 2.16 b	64.14 ± 4.80 a
	GM_N60	44.46 ± 6.44 a	38.55 ± 6.10 a	134.11 ± 6.10 a	63.14 ± 6.00 a
	WF_N100	33.24 ± 2.61 a	19.19 ± 1.93 b	114.75 ± 1.93 b	61.09 ± 0.92 a
	GM_N100	47.57 ± 7.29 a	29.69 ± 8.11 ab	125.25 ± 8.11 ab	61.12 ± 2.31 a
GM		51.08 ± 4.01 **	32.95 ± 3.19 **	128.51 ± 3.19 **	60.33 ± 2.30 *
WF		34.38 ± 2.84	23.77 ± 1.44	119.33 ± 1.44	63.89 ± 1.50

Note: Data are presented as the mean ± standard error of 4 replicates. Different letters within the same column indicate significant differences (*p* < 0.05); * represents *p* < 0.05; ** represents *p* < 0.01. The same applies below.

**Table 5:** K fertilizer use efficiency under co-utilization of milk vetch and rice straw.

Treatment		K Fertilizer Recovery Efficiency (%)	K Fertilizer Agronomic Efficiency (kg/kg)	K Fertilizer Partial Productivity (kg/kg)	K Fertilizer Harvest Index (%)
without S	WF_N0	30.53 ± 11.66 b	8.52 ± 4.23 b	80.19 ± 4.23 a	11.00 ± 1.60 a
	GM_N0	57.37 ± 3.09 a	12.41 ± 3.39 ab	80.69 ± 4.80 b	8.93 ± 1.12 a
	WF_N60	73.93 ± 8.80 a	17.58 ± 2.86 ab	89.25 ± 2.86 ab	8.98 ± 0.51 a
	GM_N60	82.37 ± 5.29 a	23.58 ± 4.69 a	95.25 ± 4.69 a	8.47 ± 1.19 a
	WF_N100	64.17 ± 5.26 a	21.75 ± 1.20 a	93.42 ± 1.20 a	7.98 ± 0.49 a
	GM_N100	67.47 ± 6.16 a	24.08 ± 5.10 a	95.75 ± 5.10 a	8.49 ± 0.73 a
with S	WF_N0	49.13 ± 4.50 b	9.71 ± 4.69 c	81.38 ± 4.69 c	9.21 ± 1.04 a
	GM_N0	69.10 ± 6.35 ab	14.58 ± 0.61 bc	82.69 ± 3.61 bc	8.78 ± 0.45 a
	WF_N60	71.25 ± 10.62 a	17.58 ± 1.62 bc	89.25 ± 1.62 bc	8.72 ± 1.27 a
	GM_N60	71.82 ± 7.01 a	28.91 ± 4.58 a	100.58 ± 4.58 a	9.85 ± 0.70 a
	WF_N100	70.24 ± 5.13 a	14.39 ± 1.45 bc	86.06 ± 1.45 bc	7.63 ± 0.26 a
	GM_N100	68.53 ± 5.24 ab	22.27 ± 6.08 ab	93.94 ± 6.08 ab	9.18 ± 0.82 a
GM		72.55 ± 3.07 *	24.71 ± 2.39 **	96.38 ± 2.39 *	9.00 ± 0.42 ^n.s.^
WF		69.90 ± 3.61	17.82 ± 1.08	89.49 ± 1.085	8.33 ± 0.36

Note: Data are presented as the mean ± standard error of 4 replicates. Different letters within the same column indicate significant differences (*p* < 0.05); * represents *p* < 0.05; ** represents *p* < 0.01. The same applies below.

**Table 6:** Soil nutrient content under co-utilization of milk vetch and rice straw treatments.

Treatment	SOM (g/kg)	TN (g/kg)	TP (g/kg)	TK (g/kg)	NH_4_^+^-N (mg/kg)	NO_3_^−^N (mg/kg)	AP (mg/kg)	AK (mg/kg)
WF_N0	20.26 ± 1.97 ^a^	1.42 ± 0.08 ^a^	0.44 ± 0.01 ^ab^	6.96 ± 0.1 ^c^	1.9 ± 0.5 ^b^	3.1 ± 0.6 ^a^	18 ± 2 ^a^	79 ± 3 ^a^
GM_N0	20.97 ± 1.42 ^a^	1.21 ± 0.04 ^ab^	0.45 ± 0.02 ^ab^	9.70 ± 1.04 ^b^	2.0 ± 0.2 ^b^	4.7 ± 0.6 ^a^	19 ± 1 ^a^	84 ± 5 ^a^
WFS_N0	21.96 ± 1.69 ^a^	1.22 ± 0.05 ^ab^	0.44 ± 0.01 ^ab^	10.28 ± 0.26 ^ab^	1.4 ± 0.1 ^b^	3.8 ± 0.8 ^a^	22 ± 3 ^a^	83 ± 2 ^a^
GMS_N0	22.61 ± 1.57 ^a^	1.30 ± 0.05 ^ab^	0.48 ± 0.01 ^a^	10.88 ± 0.12 ^a^	1.9 ± 0.1 ^b^	4.3 ± 1.0 ^a^	22 ± 2 ^a^	83 ± 2 ^a^
WF_N60	22.05 ± 0.56 ^a^	1.15 ± 0.03 ^b^	0.45 ± 0.01 ^ab^	6.65 ± 0.19 ^c^	2.1 ± 0.2 ^b^	3.3 ± 0.7 ^a^	22 ± 3 ^a^	77 ± 2 ^a^
GM_N60	21.63 ± 1.19 ^a^	1.17 ± 0.11 ^b^	0.41 ± 0.01 ^b^	6.60 ± 0.07 ^c^	1.9 ± 0.2 ^b^	3.1 ± 0.6 ^a^	19 ± 2 ^a^	74 ± 2 ^a^
WFS_N60	21.63 ± 0.35 ^a^	1.24 ± 0.04 ^ab^	0.44 ± 0.007 ^ab^	6.70 ± 0.17 ^c^	2.2 ± 0.2 ^b^	2.6 ± 0.7 ^a^	21 ± 1 ^a^	79 ± 4 ^a^
GMS_N60	23.16 ± 1.62 ^a^	1.29 ± 0.11 ^ab^	0.44 ± 0.01 ^ab^	6.68 ± 0.06 ^c^	1.7 ± 0.1 ^b^	3.3 ± 0.7 ^a^	17 ± 1 ^a^	79 ± 4 ^a^
WF_N100	21.53 ± 1.61 ^a^	1.12 ± 0.08 ^b^	0.44 ± 0.02 ^ab^	6.63 ± 0.13 ^c^	1.9 ± 0.4 ^b^	2.9 ± 0.6 ^a^	19 ± 2 ^a^	81 ± 4 ^a^
GM_N100	21.48 ± 0.54 ^a^	1.15 ± 0.05 ^b^	0.44 ± 0.02 ^ab^	6.49 ± 0.19 ^c^	4.4 ± 1.4 ^a^	3.2 ± 0.4 ^a^	18 ± 2 ^a^	82 ± 4 ^a^
WFS_N100	23.16 ± 1.51 ^a^	1.17 ± 0.05 ^b^	0.43 ± 0.02 ^ab^	6.61 ± 0.04 ^c^	1.4 ± 0.2 ^b^	2.8 ± 0.6 ^a^	18 ± 2 ^a^	86 ± 8 ^a^
GMS_N100	23.57 ± 0.74 ^a^	1.23 ± 0.05 ^ab^	0.43 ± 0.02 ^ab^	6.53 ± 0.06 ^c^	2.4 ± 0.5 ^b^	3.0 ± 0.4 ^a^	17 ± 2 ^a^	81 ± 3 ^a^

Note: SOM, TN, TP, TK, NH_4_^+^-N, NO_3_^−^-N, AP, and AK represent soil organic matter, total nitrogen, total phosphorus, total potassium, ammonium nitrogen, nitrate nitrogen, available phosphorus, and soil available potassium, respectively. Data are presented as the mean ± standard error of 4 replicates. Different letters within the same column indicate significant differences (*p* < 0.05). The same applies below.

## Data Availability

The datasets generated for this study are available upon request to the corresponding author.

## References

[1] Alengebawy A., Ran Y., Ghimire N., Osman A.I., Ai P. (2023). Rice straw for energy and value-added products in China: A review. Environ. Chem. Lett..

[2] National Bureau of Statistics of China (2020). China Statistical Yearbook.

[3] Chen X., Cui Z., Fan M., Vitousek P., Zhang F., Yan X., Yang J. (2014). Producing more grain with lower environmental costs. Nature.

[4] Zhang X., Davidson E.A., Mauzerall D.L., Searchinger T.D., Dumas P., Shen Y. (2015). Managing nitrogen for sustainable development. Nature.

[5] Fowler D., Pyle J.A., Raven J.A., Sutton M.A. (2013). The global nitrogen cycle in the twenty-first century: Introduction. Philos. Trans. R. Soc. Lond. B.

[6] Bouwman A.F., Beusen A.H.W., Billen G. (2009). Human alteration of the global nitrogen and phosphorus soil balances for the period 1970–2050. Global Biogeochem. Cycles.

[7] De Vries W., Kros J., Kroeze C., Seitzinger S.P. (2013). Assessing planetary and regional nitrogen boundaries related to food security and adverse environmental impacts. Curr. Opin. Environ. Sustain..

[8] Fan Q., Xie J., Du J., Ge H., Wei C., Qian H., Liang H., Nie J., Hu F., Gao S. (2025). Rice straw nitrogen can be utilized by rice more efficiently when co-incorporating with milk vetch. Eur. J. Agron..

[9] Gao S.J., Zhou G.P., Chang D.N., Liang H., Nie J., Liao Y.Y., Lu Y.H., Xu C.C., Liu J., Wu J. (2023). Southern China can produce more high-quality rice with less N by green manuring. Resour. Conserv. Recycl..

[10] Yang Z.P., Xu M.G., Zheng S.X., Nie J., Gao J.S., Liao Y.L., Xie J. (2012). Effects of Long-Term Winter Planted Green Manure on Physical Properties of Reddish Paddy Soil Under a Double-Rice Cropping System. J. Integr. Agric..

[11] Li S., Nie J., Liang H., Zhou G.P., Zhang J.L., Liao Y.L., Lu Y.H., Tao Y.Y., Gao S.J., Cao W.D. (2025). Paddy fields can gain high productivity with low net global warming potential by utilizing green manure. J. Environ. Manag..

[12] Li S., Zhou G.D., Zhou G.P., Nie J., Zhang J.L., Gao S.J., Cao W.D. (2025). Rice straw returning under winter green manuring enhances soil carbon pool via stoichiometric regulation of extracellular enzymes. Soil Tillage Res..

[13] Fan Q.Y., Xu C.X., Zhang L., Xie J.C., Zhou G.P., Liu J., Hu F., Gao S.J., Cao W.D. (2023). Application of milk vetch (*Astragalus sinicus* L.) with reduced chemical fertilizer improves rice yield and nitrogen, phosphorus, and potassium use efficiency in southern China. Eur. J. Agron..

[14] Zhang Z.H., Nie J., Liang H., Wei C.L., Wang Y., Liao Y.L., Lu Y.H., Zhou G.P., Gao S.J., Cao W.D. (2023). The effects of co-utilizing green manure and rice straw on soil aggregates and soil carbon stability in a paddy soil in South China. J. Integr. Agric..

[15] Liang H., Zhou G.P., Gao S.J., Nie J., Xu C.X., Wu J., Liu C.Z., Lv Y.H., Huang Y.B., Geng M.J. (2023). Exploring site-specific N application rate to reduce N footprint and increase crop production for green manure-rice rotation system in southern China. J. Environ. Manag..

[16] Zhou G., Li G., Liang H., Liu R., Ma Z., Gao S., Chang D., Liu J., Chadwick D.R., Jones D.L. (2025). Green manure coupled with straw returning increases soil organic carbon via decreased priming effect and enhanced microbial carbon pump. Glob. Change Biol..

[17] Zhou G., Chang D., Gao S., Liang T., Liu R., Cao W. (2021). Co-incorporating leguminous green manure and rice straw drives the synergistic release of carbon and nitrogen, increases hydrolase activities, and changes the composition of main microbial groups. Biol. Fertil. Soils.

[18] Zhou G., Gao S., Chang D., Rees R.M., Cao W. (2021). Using milk vetch (*Astragalus sinicus* L.) to promote rice straw decomposition by regulating enzyme activity and bacterial community. Bioresour. Technol..

[19] Zhou G., Gao S., Chang D., Shimizu K.-Y., Cao W. (2021). Succession of fungal community and enzyme activity during the co-decomposition process of rice (*Oryza sativa* L.) straw and milk vetch (*Astragalus sinicus* L.). Waste Manag..

[20] Yang L.J., Zhang L.L., Yu C.X., Li D.P., Gong P., Xue Y., Song Y.C., Cui Y.L., Doane T.A., Wu Z.J. (2017). Nitrogen fertilizer and straw applications affect uptake of 13C, 15N-glycine by soil microorganisms in wheat growth stages. PLoS ONE.

[21] Zhu L.X., Xiao Q., Shen Y.F., Li S.Q. (2017). Effects of biochar and maize straw on the short-term carbon and nitrogen dynamics in a cultivated silty loam in China. Environ. Sci. Pollut. Res..

[22] Zhou G., Cao W., Bai J.S., Xu C.X., Zeng N.H., Gao S.J. (2019). Non-additive responses of soil C and N to rice straw and hairy vetch (*Vicia villosa* Roth L.) mixtures in a paddy soil. Plant Soil..

[23] Yang L., Zhou X., Liao Y., Lu Y., Nie J., Cao W. (2019). Co-incorporation of rice straw and green manure benefits rice yield and nutrient uptake. Crop Sci..

[24] Kaewpradit W., Toomsan B., Cadisch G., Vityakon P., Limpinuntana V., Saenjan P., Jogloy S., Patanothai A. (2009). Mixing groundnut residues and rice straw to improve rice yield and N use efficiency. Field Crops Res..

[25] Blonska E., Piaszczyk W., Staszel K., Lasota J. (2021). Enzymatic activity of soils and soil organic matter stabilization as an effect of components released from the decomposition of litter. Appl. Soil Ecol..

[26] Hoorens B., Aerts R., Stroetenga M. (2002). Litter quality and interactive effects in litter mixtures: More negative interactions under elevated CO_2_?. J. Ecol..

[27] Pramanik P., Haque M.M., Kim S.Y., Kim P.J. (2014). C and N accumulations in soil aggregates determine nitrous oxide emissions from cover crop treated rice paddy soils during fallow season. Sci. Total Environ..

[28] Yang L. (2019). Effects of *Astragalus sinicus* L. and Rice Straw Co-Application on Fertilizer Reduction and Nitrogen Fixation Regulation Mechanism.

[29] Bao S.D. (2000). Soil Agrochemical Analysis.

[30] Gao S.J., Zhou G.P., Cao W.D. (2020). Increased Yield and Fertilizer-saving Effects of *Astragalus sinicus* L. as a Winter Green Manure in Southern Paddy Fields. Plant Nutr. Fertil. Sci..

[31] Zhou G.P., Xie Z.J., Cao W.D., Xu C.X., Bai J.S., Zeng N.H., Gao S.J., Yang L. (2017). Improving Soil Fertility and Crop Yield via Combined Incorporation of Rice Straw Stubble and *Astragalus sinicus* L. Trans. Chin. Soc. Agric. Eng..

[32] Cai S., Shi H., Pan X.H., Chen Y., Xu T., Wan S.Y. (2020). Effects of Combined Green Manure and Rice Straw Return on Photosynthetic Characteristics, Nutrient Absorption, and Yield Quality of Machine-transplanted Rice. J. Jiangxi Agric. Univ..

[33] Cai S., Shi H., Pan X.H., Xu T., Xie H.W., Liu F.P., Cao N. (2019). Effects of Combined Green Manure and Rice Straw Return on Growth and Yield of Double-cropped Rice. J. Jiangxi Agric. Univ..

[34] Li S., Liang H., Wang Y., Zhang Z.H., Zhang L., Zhou G.P., Gao S.J., Cao W.D. (2022). Responses of functional genes involved in nitrogen cycling to green manuring in different paddy soils in south China. Plant Soil.

[35] Wang H., Zhang L., Chang D.N., Zhou G.P., Gao S.J., Zeng N.H., Nie L.P., Lv Y.H., Cao W.D. (2022). Effects of reducing nitrogen fertilizer application rates on nutrient uptake and translocation in rice in the south Henan alfalfa-rice rotation zone. Plant Nutr. Fertil. Sci..

[36] Huang J., Gao J.S., Liu S.J., Cao W.D., Zhang Y.Z. (2013). Effects of Winter-sown *Astragalus sinicus* L. on Rice Yield and Nutrient Absorption. Chin. J. Soil Fertil..

[37] Wu W.G., Zhang S.H., Zhao J.J., Wu G.C., Li Z.F., Xia J.F. (2007). Effects of Nitrogen Fertilizer Management on Nitrogen Uptake and Utilization and Yield of Double-cropped Rice at the Northern Margin. Plant Nutr. Fertil. Sci..

[38] Tang H.M., Xiao X.P., Li C., Tang W.G., Guo L.J., Cheng K.K., Li W.Y. (2019). Effects of Different Soil Tillage Systems on Nutrient Accumulation and Translocation in Double-cropped Rice. J. Nanjing Agric. Univ..

[39] Fontaine S., Mariotti A., Abbadie L. (2003). The priming effect of organic matter: A question of microbial competition. Soil Biol. Biochem..

[40] Lal R. (2007). Soil science and the carbon civilization. Soil Sci. Soc. Am. J..

[41] Chatterjee A. (2013). Annual crop residue production and nutrient replacement costs for bioenergy feedstock production in United States. Agron. J..

[42] Wang Q., Wang Y., Wang Q., Liu J. (2014). Impacts of 9 years of a new conservational agricultural management on soil organic carbon fractions. Soil Tillage Res..

[43] Schwendener C.M., Lehmann J., de Camargo P.B., Luizão R.C.C., Fernandes E.C.M. (2005). Nitrogen transfer between high- and low-quality leaves on a nutrient-poor Oxisol determined by 15N enrichment. Soil Biol. Biochem..

[44] Nie J., Yi L., Xu H., Liu Z., Zeng Z., Dijkstra P., Koch G.W., Hungate B.A., Zhu B. (2019). Leguminous cover crop *Astragalus sinicus* enhances grain yields and nitrogen use efficiency through increased tillering in an intensive double-cropping rice system in southern China. Agronomy.

[45] Liu C.Z., Chang D.N., Li B.Y., Cao W.D., Lü Y.H., Pan Z.L. (2017). Effects of green manure and fertilizer application on active organic carbon and nitrogen in paddy soils. Acta Pedol. Sin..

[46] Meng X.T., Li Y.Y., Zhang Y., Yao H.Y. (2019). Green manure application improves rice growth and urea nitrogen use efficiency assessed using 15N labeling. Soil Sci. Plant Nutr..

[47] Liang H., Li S., Zhang L., Xu C.X., Lv Y.H., Gao S.J., Cao W.D. (2022). Long-term green manuring enhances crop N uptake and reduces N losses in rice production system. Soil Tillage Res..

[48] Schoenholtz S.H., Miegroet H.V., Burger J.A. (2000). A review of chemical and physical properties as indicators of forest soil quality: Challenges and opportunities. For. Ecol. Manag..

[49] Li L., Zhu H.H., Su Y.R., Xiao H.A., Huang D.Y., Wu J.S. (2009). Effects of Straw Return and Soil Relocation on Soil Organic Carbon and Its Active Fractions in Hilly Red Soil Farmland. Chin. J. Agric. Sci..

[50] Wang X.D., Shi X.J., Song G.Y. (2005). Long-term effect of rice straw return on soil fertility and productivity of purple paddy soil. Plant Nutr. Fertil. Sci..

[51] Wang Q.J., Bai Y.H., Gao H.W., He J., Chen H., Chesney R.C., Kuhn N.J., Li H.W. (2008). Soil chemical properties and microbial biomass after 16 years of no-tillage farming on the Loess Plateau, China. Geoderma.

[52] Singh G., Jalota S.K., Singh Y. (2007). Manuring and residue management effects on physical properties of a soil under the rice–wheat system in Punjab, India. Soil Tillage Res..

[53] Wang J., Zhang F., Zhang X., Cao Y. (2000). Release of potassium from K-bearing minerals: Effect of plant roots under P deficiency. Nutr. Cycl. Agroecosyst..

[54] Appelt H., Coleman N.T., Pratt P.F. (1975). Interactions between organic compounds, minerals, and in volcanic-ash-derived soils: Effects of organic compounds on the adsorption of phosphate. Soil Sci. Soc. Am. J..

[55] Zhang F., Ma J., Cao Y. (1997). Phosphorus deficiency enhances root exudation of low-molecular weight organic acids and utilization of sparingly soluble inorganic phosphates by radish (*Raphanus sativus* L.) and rape (*Brassica napus* L.) plants. Plant Soil.

[56] Wei L., Liu S., Hussain L., Wu Z., Qin X., Li X. (2016). Greenhouse gas emissions, soil quality, and crop productivity from a mono-rice cultivation system as influenced by fallow season straw management. Environ. Sci. Pollut. Res..

[57] Gao J.S., Huang J., Yang Z.C., Cao W.D., Zhang H.M., Gao P., Gao X.C. (2020). Combined application of green manure and rice straw increases soil organic matter and stabilizes nitrogen supply. Plant Nutr. Fertil. Sci..

[58] Arlauskiene A., Slepetiene A., Liaudanskiene I., Sarunaite L., Amaleviciute K., Velykis A. (2015). The influence of short-lived legume swards and straw on soil humic substances in a clay loam cambisol. Fresenius Environ. Bull..

[59] Thorup-Kristensen K., Halberg N., Nicolaisen M., Olesen J.E., Crews T.E., Hinsinger P., Kirkegaard J., Pierret A., Dresboll D.B. (2020). Digging Deeper for Agricultural Resources, the Value of Deep Rooting. Trends Plant Sci..

[60] Guo N., Zhang S.N., Gu M.J., Xu G.H. (2021). Function, transport, and regulation of amino acids: What is missing in rice?. Crop J..

